# Evaluation of the Community Urgent Eyecare Service in Greater Manchester, Including the Impact of Independent Prescribing

**DOI:** 10.1007/s44402-026-00137-w

**Published:** 2026-07-21

**Authors:** Aoife L. Y. Quah, Amy Hughes, Wendy Craven, Helen Wilson, Emma Williams, Felipe Dhawahir-Scala, Patrick J. G. Gunn, Robert A. Harper

**Affiliations:** 1 The School of Medicine, Dentistry and Biomedical Sciences, Faculty of Medicine, Health and Life Sciences, Queen’s University Belfast, Belfast, UK; 2 Division of Pharmacy and Optometry, Faculty of Biology, Medicine and Health Sciences, School of Health Sciences, The University of Manchester, Manchester, UK; 3 Primary Eyecare Services, Manchester, UK; 4 Manchester Royal Eye Hospital and Manchester Academic Health Sciences Centre, Manchester University NHS Foundation Trust, Manchester, UK

**Keywords:** Independent prescribing, Urgent eye care, Community optometry

## Abstract

**Purpose:**

Community Urgent Eyecare Services (CUES) utilises primary care optometrists to reduce urgent eyecare demand within ophthalmology and general practice (GP). This large-scale study evaluated CUES in Greater Manchester (GM), including assessing the embedded Independent Prescribing (IP) pathway.

**Methods:**

This retrospective, GM-wide analysis evaluated CUES data from 1st January to 31st December 2024. Primary care data included patient pathways, case mix and IP outcomes. Index of Multiple Deprivation (IMD) deciles were assigned to explore socioeconomic associations with CUES access. Secondary care data included sampling CUES referrals to Manchester Royal Eye Hospital (MREH), assessing diagnostic agreement with Eye Emergency Department (EED) clinicians and determining the proportion of cases potentially manageable by IP optometrists in primary care.

**Results:**

In 2024, GM CUES managed 54,994 patients; 69.9% were self-presenting, and 78.2% were assessed, managed and discharged without referral. Furthermore, 5.2% of cases were referred to General Medical Practitioners (GPs), resulting in 83.4% overall being retained in primary care. Patients in the most deprived IMD decile attended more frequently. IP-episode discharge rate was significantly higher (87.5%, *p* = 0.000001) than non-IP episodes (78.1%). In the referred sub-sample, diagnostic agreement with EED clinicians was 75.0%, with 40.0% of these cases being potentially manageable within primary care had IP capacity been available. Primary care National Health Service prescriptions (FP10 prescriptions) were issued for 73.0% (*N* = 362) of IP cases with corticosteroids (43.7%, *N* = 190), antibiotics (14.3%, *N* = 62) and antimuscarinics (12.4%, *N* = 54) being the most common.

**Conclusion:**

GM CUES managed and discharged most cases in primary care, reducing GP and EED demands. Greater IP optometrist availability could broaden CUES’ scope and increase effectiveness.

Key Points

- This large-scale evaluation of the Community Urgent Eyecare Service in Greater Manchester finds that Community Urgent Eyecare Service optometrists assess, manage and discharge a high volume of patients.
- This study reports a higher discharge rate for Independent Prescriber optometrist-managed episodes than for non-Independent Prescriber optometrist-managed episodes, with many cases suitable for Independent Prescriber management being referred to hospital eye services.
- Increasing Independent Prescriber optometrist capacity within the Community Urgent Eyecare Service appears likely to allow greater numbers of Community Urgent Eyecare Service patients to be retained in primary care.

## Introduction

Emergency departments (EDs) and emergency eye departments (EEDs) face increasing demands, with ocular presentations accounting for ~6% of general ED attendances [1, 2]. The annual EED walk-in rate has been estimated as 31.0 per 1000 new patients [3]. However, the Royal College of Ophthalmologists’ guidance recognises that many conditions presenting to EED, despite being urgent, are not typically emergencies [4]. In response to growing pressures, there has been a shift towards delivering urgent eyecare through primary care optometry [5].

**Fig. 1:**
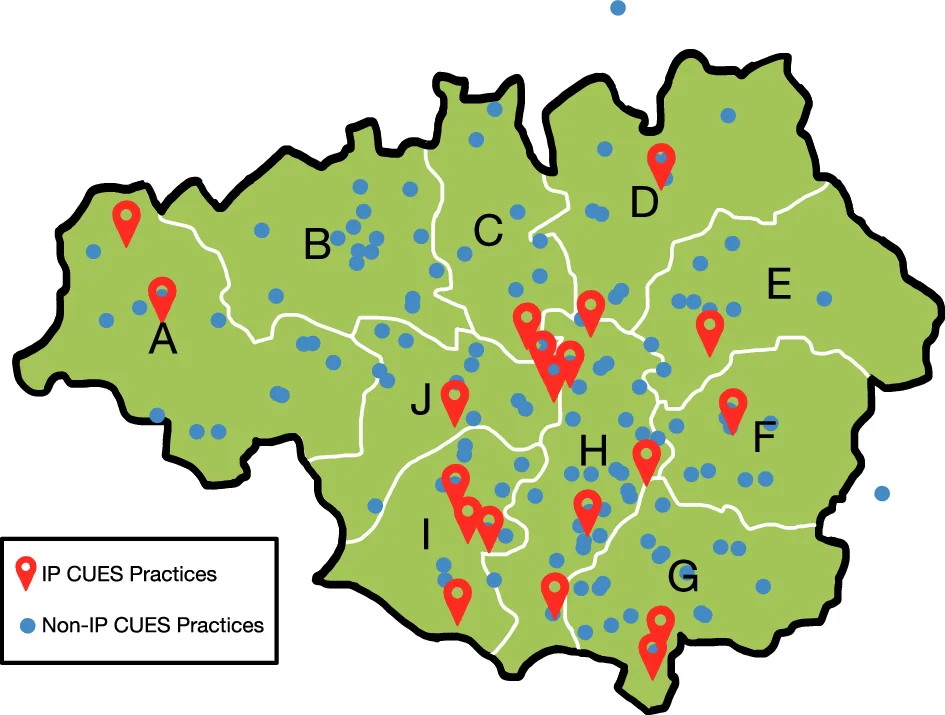
Map marking the Community Urgent Eyecare Service (CUES) practices in Greater Manchester and highlighting those managing Independent Prescriber (IP) episodes. Individual boroughs are marked as follows: A = Wigan, B = Bolton, C = Bury, D = Rochdale, E = Oldham, F = Tameside, G = Stockport, H = Manchester, I = Trafford, J = Salford. Developed using Kar’s image [40]. Number of non-IP CUES practices = 198, 89.6%; Number of IP CUES practices = 23, 10.4%.

The Greater Manchester (GM) COVID-19 Urgent Eyecare Service (Community Urgent Eyecare Service (CUES)) was introduced in April 2020 to reduce hospital or face-to-face attendances during the COVID-19 pandemic [6]. CUES aims to alleviate pressures on General Medical Practitioners (GPs and EDs) by utilising primary care optometrist skills and providing local and timely care for urgent eye presentations. Since renamed the CUES in 2022, its primary aims remain [7]. Across England, Clinical Commissioning Groups (since regrouped into Integrated Care Boards) were urged to commission CUES and, as of 2022, surveys indicated that 43% of Clinical Commissioning Groups had CUES access [8–10]. Within GM, CUES is now commissioned across all 10 Integrated Care Board localities (Fig. 1) [11].

An early evaluation by Kanabar et al. [12] indicated that CUES retained 85.7% of cases within primary care (discharged by CUES or referred to GPs) with a contemporaneous reduction in EED attendances, and 63.9% of CUES-referred cases were in ‘agreement’ or ‘partial agreement’ with EED. However, this evaluation was conducted during COVID-19, prior to the GM-wide rollout, and when Independent Prescriber (IP) optometrist pathways were limited. The present study aimed to undertake a large-scale, year-long updated evaluation of GM CUES post-COVID-19, considering the region-wide service expansion and IP pathway developments.

## Methods

### Primary Care Data

Routine GM CUES data from January 1st to December 31st, 2024, were collected via the Primary Eyecare Services Information Technology (IT) Platform and collated with patient-identifiable data removed. Eligible cases for inclusion were those assessed within GM CUES within the 12-month study period. Parameters included patient demographics, source of signposting to CUES, summary of symptoms, appointment type, diagnoses and outcomes.

Presenting symptoms were a simplified categorisation based on CUES triaging forms, where symptoms were marked yes/no for: ‘foreign body’; ‘problem with vision’; ‘red, painful eye’; or ‘flashes and floaters’.

**Fig. 2:**
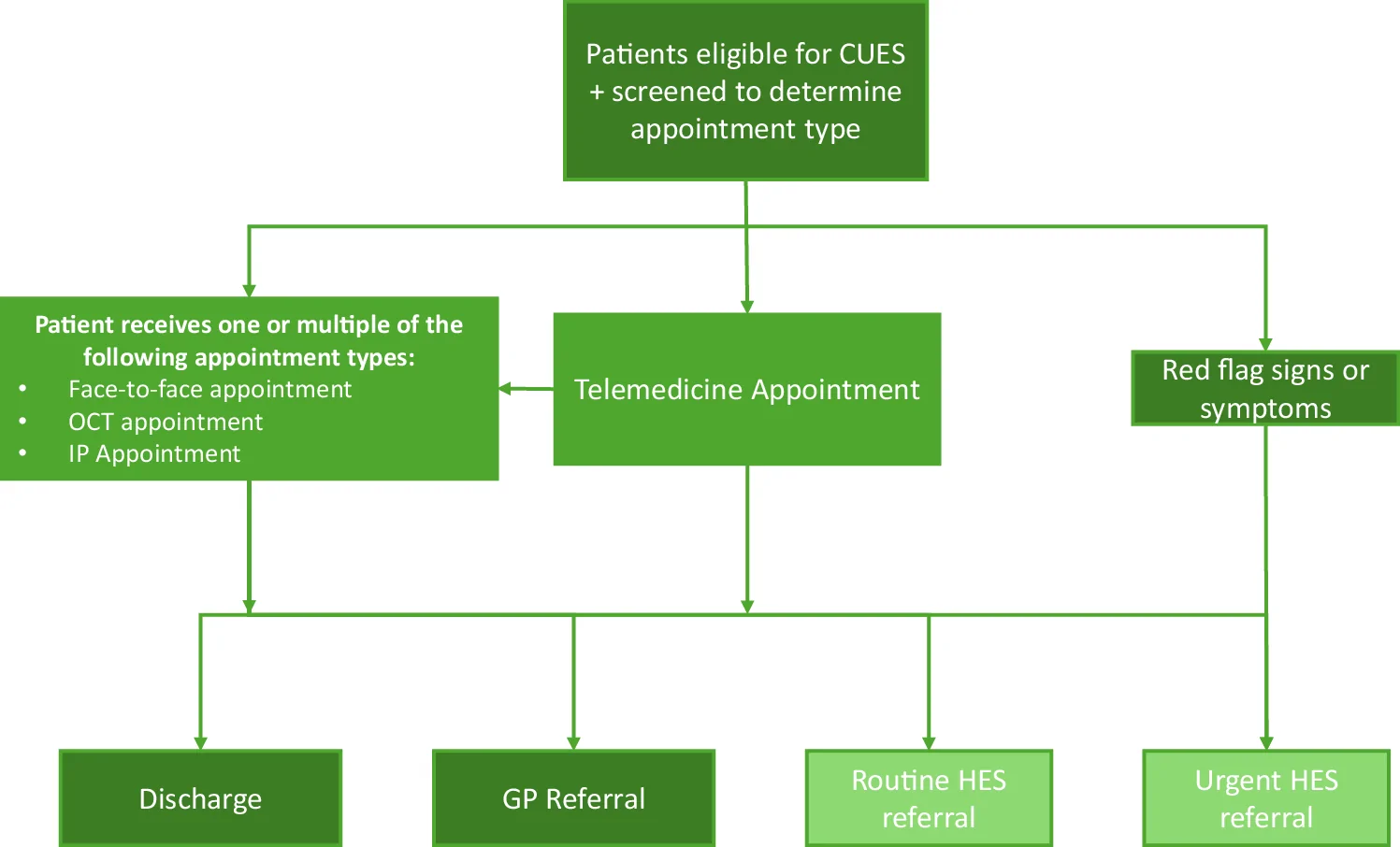
Flowchart illustrating routes of patient flow through Community Urgent Eyecare Service (CUES) in Greater Manchester, including appointment types and outcomes. GP general medical practitioner, HES hospital eye service, IP independent prescribing, OCT optical coherence tomography.

Figure 2 outlines the CUES pathway mapping appointment types and outcomes. Appointment types were defined as telemedicine, face-to-face, optical coherence tomography (OCT) (i.e., appointment including OCT where the patient presented with central vision issues), and IP (i.e., appointment where signs and/or symptoms indicated an appointment with an IP optometrist). In 2022, the CUES pathway was updated to remove mandated telemedicine prior to face-to-face appointments. As the pathway change was adopted at different times across England, the national IT platform continued to reflect the original 2020 pathway, creating potential ambiguity around whether episodes recorded as telemedicine may incorrectly include patients proceeding directly to face-to-face assessments. Where ambiguity existed, assumptions were applied using relevant clinical indicators recorded to reflect face-to-face episodes (e.g., recorded dilation, drop installation, intraocular pressure measurements, visual field testing or posterior eye examination). If a telemedicine and face-to-face appointment were recorded on the same day, the consultation was assumed to be face-to-face. Follow-up appointments were defined as planned or ad hoc re-presentations for the same issue within 3 months of initial assessment. Outcomes included management and discharge, referral to GP and referral to the hospital eye service (HES) (urgent or routine).

For each patient’s residential part-postcode, respective indices of multiple deprivation (IMD) deciles were assigned [13]. Further, all patients accessing CUES are asked for consent to receive a short message service text or email patient-reported experience measure (PREM) questionnaire, with consenting patients asked to rate their experience on a 1–7-point scale (7 being excellent), whether they would recommend the service to friends or family and about alternative care-seeking behaviour had CUES been unavailable [14].

From the 12-month GM-wide sample, a subgroup comprising all patients from the first week of each month plus all IP cases was taken to assess case mix with more granularity, i.e., the proportions of different conditions diagnosed. Diagnoses were reviewed and matched to the set conditions list introduced in 2025 and categorised by anatomical area, aligned with a previous CUES evaluation [12].

IP cases were defined as those presenting to and managed by an IP-qualified optometrist applying their prescribing qualification to make clinical decisions, or cases referred to them by a non-IP colleague seeking an IP optometrist’s opinion. IP-qualified optometrists could also examine patients through core assessments, where their prescribing qualification was unlikely to be required, with these encounters not being classified as IP cases. Conditions diagnosed during IP and non-IP episodes were quantified and compared. National Health Service (NHS)-funded primary care FP10 and private prescriptions were evaluated to quantify prescribing activity by medication, grouped as per the British National Formulary [15]. If multiple medications were prescribed on one FP10, each was recorded separately.

### Secondary Care Data

At the Manchester Royal Eye Hospital (MREH), GM’s leading HES provider, 120 CUES referrals to EED were systematically sampled from 10 days across each month in 2024. Cases were followed up in MREH’s electronic records to determine the subsequent EED diagnosis and management. Two experienced EED optometrists independently rated all 120 cases as being in ‘agreement’, ‘partial agreement’ or ‘disagreement’ between the CUES and EED diagnoses. The agreement included cases where the optometrist correctly recognised the precise diagnosis. Partial agreement included cases where the CUES diagnosis was partially correct but amended following EED assessment (e.g., CUES diagnosis of anterior uveitis and HES diagnosis of iridocyclitis). Disagreement included CUES diagnoses proven incorrect at EED, for example, a referred retinal tear later proven to be only a routine posterior vitreous detachment without evidence for a tear. Disagreements between raters were resolved via a consensus meeting with a third clinician (Clinical Lead ophthalmologist for EED). Chi-squared testing was used to compare diagnostic agreement between CUES and EED clinicians for both IP and non-IP optometrist cases. Through the same process, the 120 referred cases were classified as ‘yes’ or ‘no’ in response to the question ‘Could this case have been managed in primary care by an IP optometrist?’ using the raters’ knowledge on NHS GM eyecare pathways and relevant College of Optometrists’ Clinical Management Guidelines. A not applicable (N/A) category was assigned to cases where prescribing, and thus IP optometrist involvement, was irrelevant (e.g., flashes and floaters).

### Ethical Considerations

This evaluation utilised routinely collected, anonymised information held by Primary Eyecare Services. Following the University of Manchester Ethics Decision Tool and the Health Research Authority Toolkit, this project was deemed not to require formal ethical approval [16, 17]. The project was registered as an audit within MREH (reference number 12349), and AQ, the primary researcher, held a contemporaneous honorary contract while working under the supervision of the authors. While patients were not involved in the design of this research, their views have been captured within the evaluation using PREMs. Further, we confirm that the Standards for Quality Improvement Reporting Excellence (SQUIRE) checklist was used as the quality framework for informing the completeness of this work and the write-up [18].

## Results

### Patient Journey

In 2024, GM CUES managed 54,994 cases, with a mean age of 50.4 years (range 0–101, SD = 23.0 years) and a higher proportion of females (61.1%). Entrance into CUES included: 69.9% (*N* = 38,458) self-referrals; 11.7% (*N* = 6450) GP staff referrals (having not seen a GP); 6.6% (*N* = 3628) from GPs; 3.0% (*N* = 1670) from pharmacists; 2.9% (*N* = 1580) from NHS111 service (a free UK NHS 24 h helpline for non-emergency medical conditions); 1.7% (*N* = 923) from non-CUES providing optometrists; 1.2% (*N* = 648) from hospital eye clinics and 1.1% (*N* = 608) from General Ophthalmic Services sight testing. The remaining 1.9% (*N* = 1029) came from EDs, GP out of hours, referrals following private eye examinations, primary care ophthalmology clinics, urgent treatment centres, minor injuries units and others.

Overall, CUES optometrists assessed, managed and discharged 42,997 (78.2%) patients without need for onward referral. A further 5.2% (*N* = 2861) were referred to GPs. Overall, 83.4% (*N* = 45,858) were retained in primary care. HES received 6664 (12.1%) urgent and 2472 (4.5%) routine referrals.

### Appointment Types

**Fig. 3:**
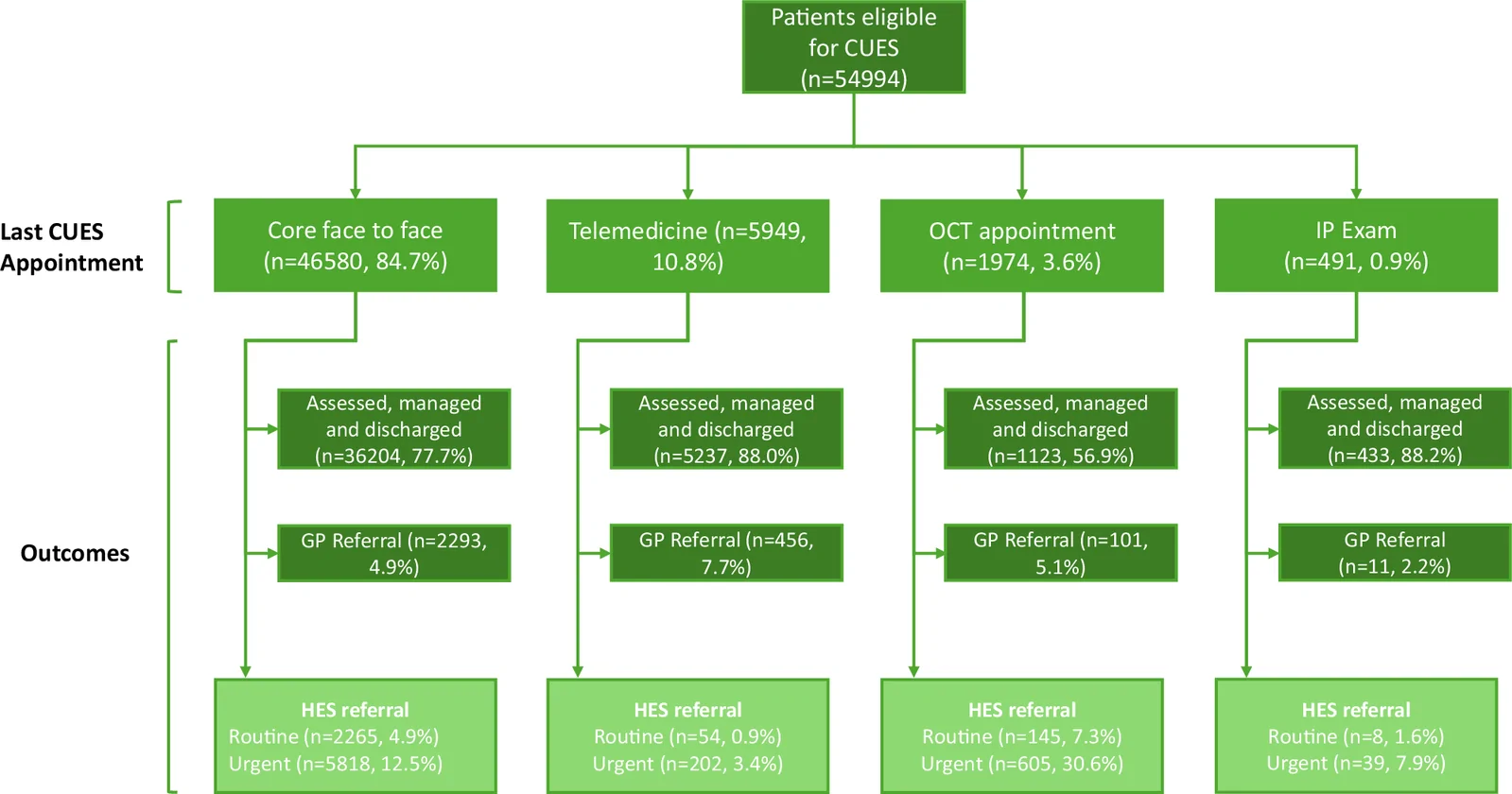
Flowchart mapping patient flow through the service based on outcomes following each patient’s final appointment within the Community Urgent Eyecare Service (CUES). GP general medical practitioner, IP independent prescribing, HES hospital eye service, OCT optical coherence tomography. Labelled with case numbers (*N*) and corresponding percentage of total case number (*x*%) (e.g., *N*, *x*%).

Figure 3 maps patients’ flow through CUES, based on outcomes following patients’ final appointment types. Additionally, 87.3% of cases (*N* = 48,026) received at least one core face-to-face appointment and 2278 (4.1%) of cases received at least one follow-up appointment.

A total of 8658 patients (~16% based on assumptions detailed in the methods) were estimated to have had at least one telemedicine consultation, with 5949 (10.8% of total) managed entirely by telemedicine. Of these cases managed entirely by telemedicine, 88.0% (*N* = 5237) were discharged, 456 (7.7%) were referred to GPs and 256 (4.3%) were referred to HES (202 urgent referrals, 3.4%; 54 routine referrals, 0.9%).

### Symptoms and Conditions

Key symptoms presenting at triaging included 66.4% (*N* = 36,506) with a painful red eye, 17.1% (*N* = 9415) reporting flashes/floaters, 12.3% (*N* = 6737) reporting problems with their vision and 3.8% (*N* = 2073) with foreign body symptoms.

The subgroup comprising all cases presenting in the first week of each month plus the total annual IP cases captured 13,176 cases (496 IP cases and 12,680 non-IP cases). The frequently diagnosed/tentatively diagnosed conditions for both IP and non-IP assessments are listed in Tables 1 and 2. Eyelid conditions were most common in non-IP cases (*N* = 2888, 22.8% of non-IP subgroup total case number), whereas conjunctival conditions were most prevalent amongst IP cases (28.8% of total IP cases, *N* = 143). The most commonly urgently referred conditions were: retinal detachment, tear or hole (*N* = 173, 10.7% of all urgent HES referrals); anterior uveitis/iritis (*N* = 141, 8.7%); corneal ulcer (*N* = 105, 6.49%); foreign body (corneal, subtarsal, conjunctival) (*N* = 94, 5.81%) and wet age-related macular degeneration (*N* = 81, 5.01%).

**Table 1:** Most common tentatively diagnosed conditions within the Community Urgent Eyecare Service (CUES) for IP and non-IP cases.

Conditions diagnosed	Number of cases (% of column total)		
	Whole subgroup	Non-IP cases	IP cases
Dry eye disease (Keratoconjunctivitis Sicca)	2023 (15.4)	1997 (15.7)	26 (5.2)
Vitreous Conditions (Degeneration/ PVD, No PVD/retinal detachment/tear/ hole signs)	1560 (11.8)	1560 (12.3)	0 (0.0)
Conjunctivitis (Bacterial, Viral (incl. follicular))	998 (7.6)	940 (7.4)	58 (11.7)
Allergic conjunctivitis	746 (5.7)	678 (5.3)	68 (13.7)
Subconjunctival haemorrhage	658 (5.0)	657 (5.2)	1 (0.2)
External hordeolum	557 (4.2)	524 (4.1)	33 (6.7)
Chalazion	542 (4.1)	528 (4.2)	14 (2.8)
Blepharitis	523 (4.0)	499 (3.9)	24 (4.8)
No pathology identified	447 (3.4)	443 (3.5)	4 (0.8)
Migraine with visual aura, Ocular migraine	430 (3.3)	430 (3.4)	0 (0.0)
Corneal abrasion	351 (2.7)	332 (2.6)	19 (3.8)
Foreign body (corneal, subtarsal, conjunctival)	326 (2.5)	316 (2.5)	10 (2.0)
Meibomian gland dysfunction	324 (2.5)	320 (2.5)	4 (0.8)
Retinal detachment, tear or hole	239 (1.8)	239 (1.9)	0 (0.0)
Anterior uveitis, iritis	202 (1.5)	138 (1.1)	64 (12.9)
Posterior capsular opacification	200 (1.5)	200 (1.6)	0 (0.0)
Internal hordeolum	200 (1.5)	179 (1.4)	21 (4.2)
Episcleritis	194 (1.5)	168 (1.3)	26 (5.2)
Trichiasis	153 (1.2)	153 (1.2)	0 (0.0)
Preseptal cellulitis	138 (1.0)	116 (0.9)	22 (4.4)
Corneal ulcer	132 (1.0)	119 (0.9)	13 (2.6)
Wet age-related macular degeneration	89 (0.7)	89 (0.7)	0 (0.0)
Dermatitis (contact, allergic)	75 (0.6)	59 (0.5)	16 (3.2)
Marginal keratitis	39 (0.3)	29 (0.2)	10 (2.0)
Herpes simplex keratitis	28 (0.2)	20 (0.2)	8 (1.6)

*IP* Independent Prescriber, *PVD* posterior vitreous detachment.

**Table 2:** Conditions diagnosed or tentatively diagnosed in Community Urgent Eyecare Service (CUES), categorised by anatomical area for IP and non-IP-managed cases.

Anatomical area and conditions/findings included	Number of cases (% of column total)		
	Whole subgroup	Non-IP cases	IP cases
*Eyelid:* Allergy (eyelid); Blepharitis; Blepharospasm; Chalazion; Cyst (Moll/ Zeiss/dermoid/sebaceous); Dermatochalasis; Ectropion; Entropion; Epiblepharon; Eyelid haematoma/laceration/oedema; Hordeolum; Keratoacanthoma; Lagophthalmos; Malignant neoplasm of eyelid; Meibomian gland dysfunction; Molluscum contagiosum; Phthiriasis; Preseptal cellulitis; Ptosis; Pyogenic granuloma; Rosacea keratitis; Trichiasis	3021 (22.9)	2888 (22.8)	133 (26.8)
*Conjunctiva:* Conjunctivitis (bacterial/viral/atopic/allergic); Benign/malignant conjunctival lesions; Conjunctival abrasion/foreign body; Conjunctival cyst; Conjunctival oedema; Conjunctivitis medicamentosa; Conjunctivochalasis; Contact lens-associated papillary conjunctivitis; Concretions; Giant papillary conjunctivitis; Keratoconjunctivitis; Mucous fishing conjunctivitis; Ophthalmia neonatorum; Phlyctenular conjunctivitis; Pinguecula; Pingueculitis; Pterygium; Subconjunctival haemorrhage	2647 (20.1)	2504 (19.7)	143 (28.8)
*Lacrimal system:* Dacryocystitis; Dacryocystorhinostomy tube exposed; Dry eye disease; Epiphora; Keratoconjunctivitis sicca; Nasolacrimal drainage dysfunction/obstruction/stenosis/fistula; Sjögren syndrome	2092 (15.9)	2064 (16.3)	28 (5.6)
*Vitreous:* No signs of posterior vitreous detachment (PVD)/retinal detachment/tear/hole; Vitreous degeneration/floaters/pigment	1567 (11.9)	1567 (12.4)	0 (0.0)
*Cornea:* Band keratopathy; Contact lens-induced peripheral ulcer/red eye; Corneal abrasion/epithelial defect/foreign body; Corneal degeneration/dystrophy; Corneal transplant complications; Entropion with corneal involvement; Herpes zoster ophthalmicus; Keratitis (exposure/filamentary/herpes simplex/infiltrative/microbial/marginal); Keratoconus; Photokeratitis	915 (6.9)	844 (6.7)	71 (14.3)
*Optic nerve and neurological:* Amaurosis fugax; Anisocoria; Giant cell/temporal arteritis; Glaucoma (suspect/open/closed); Migraine with visual aura; Neuralgia; Nerve palsy; Non-arteritic anterior ischaemic optic neuropathy; Nystagmus; Ocular migraine; Optic disc structure anomaly; Optic neuritis/neuropathy; Papilledema; Pituitary tumour; Pupillary function anomaly; Sudden onset diplopia; Suspected neurological condition; Transient ischaemic attack; Visual field loss (non-glaucomatous)	674 (5.1)	673 (5.3)	1 (0.2)
*Retina and choroid:* Age-related macular degeneration (wet/dry/early); Choroidal naevus; Commotio retinae; Epiretinal membrane; Intracranial haemorrhage; Intraretinal/subretinal fluid; Leber’s congenital amaurosis; Macular hole; Macular oedema/maculopathy; Myelinated nerve fibres; Myopic macular degeneration; Peripheral retinal degeneration; Retinal artery/vein occlusion; (Pre-)Retinal/vitreous haemorrhage; Retinal hole/tear/detachment; Retinal lesion; Retinopathy (central serous/ diabetic/ hypertensive/ laser-induced); Retinoschisis; Roth spots; Vitreomacular traction;	603 (4.6)	602 (4.7)	1 (0.2)
No pathology identified	447 (3.4)	443 (3.5)	4 (0.8)
*Other:* Asthenopia; Chemical injury; Charles Bonnet syndrome; Dermatitis/eczema/psoriasis; Headache; Impetigo; Insect bite; Limbitis; Ocular motility disorder; Peduncular hallucinosis; Photopsia; Post-surgical/intraocular lens complication; Ruptured pupil; Side effect of botulinum toxin; Sinusitis; Sjogren syndrome; Subjective visual disturbance; Telangiectasia; Trauma; Unexplained visual loss; Vertigo	355 (2.7)	335 (2.6)	20 (4.0)
*Lens:* Cataract; posterior capsular opacification; subluxation of crystalline lens	299 (2.3)	299	0
*Sclera:* Episcleritis; scleral thinning; scleritis	225 (1.7)	195	30
*Uvea:* Anterior uveitis; iris vascular tuft; limbitis; posterior uveitis; posterior uveitis; uveal melanoma	209 (1.6)	145	64
*Refractive/Orthoptic:* Binocular vision disorder; esotropia; exotropia; hypertropia; refractive error	66 (0.5)	66	0
*Orbit:* Orbital cellulitis; orbital fracture; proptosis; thyroid eye disease	34 (0.3)	34	0

These data include cases managed by one ophthalmologist working in CUES.

*IP* independent prescribing optometrist.

### Socioeconomic Factors and CUES Access

Low IMD deciles indicate an area of greater deprivation compared to other communities. Results for attendance per IMD decile are as follows: 1st IMD *N* = 11,314 (20.6%); 2nd IMD *N* = 7328 (13.3%); 3rd IMD *N* = 5489 (10.0%); 4th IMD *N* = 4838 (8.8%); 5th IMD *N* = 4386 (8.0%); 6th IMD *N* = 3933 (7.2%); 7th IMD *N* = 4368 (8.0%); 8th IMD *N* = 4720 (8.6%); 9th IMD *N* = 4399 (8.0%) and 10th IMD *N* = 4159 (7.6%). Sixty cases were not reported since part-postcode or relevant IMD was unobtainable.

### Diagnostic Agreement

Diagnostic agreement between CUES and HES diagnoses was 75.0% (*N* = 90) in full or partial agreement; where 53.3% (*N* = 64) were in full agreement, 21.7% (*N* = 26) in partial agreement, with 25.0% (*N* = 30) in disagreement. No significant difference was found between the diagnostic agreement of IP and non-IP optometrists (df = 2, *χ*² = 0.40, *p* = 0.82, IP *N* = 10, non-IP *N* = 106). Four cases were excluded due to erroneously recorded General Optical Council (GOC) numbers on the referral forms, where IP status could not be confirmed via the GOC register. The sample was too small to conduct any meaningful statistical comparison; however, in very broad terms, corneal conditions did appear to have a higher likelihood of agreement or partial agreement between primary care and HES diagnoses.

### Independent Prescribers

Overall, 496 cases (0.9% of all CUES cases) were IP cases (i.e., assessment and management by a practitioner using their prescribing qualification); where 460 cases were managed by an IP optometrist and 36 cases were managed by a single ophthalmologist working within CUES. When the ophthalmologist’s 36 cases were excluded, outcomes following the final IP appointment are as follows: 87.5% (*N* = 398) were discharged, 1.8% (*N* = 8) were routinely referred to HES, 8.4% (*N* = 38) were urgently referred to HES, 2.4% (*N* = 11) were referred to GPs. A further five IP cases were excluded from outcome analysis, since the IP appointment was not the final consultation. Again, excluding the 36 ophthalmologist cases (to compare optometrist-optometrist rates), the IP-episode discharge rate (87.5%, *N* = 398) was significantly higher than the non-IP-episode discharge rate (78.1%, *N* = 42,432) (df = 1, *χ*² = 23.2, *p* = 0.000001).

Pharmaceutical prescriptions were issued for 73.0% (*N* = 362) of IP cases (including private prescriptions and those from ophthalmologists). Commonly issued FP10 medications included: corticosteroids (43.7%; *N* = 190); antibiotics (14.3%, *N* = 62) and antimuscarinics (12.4%, *N* = 54). Other medications included: Antihistamines *N* = 53 (12.2%); Antibacterials and fortified antibiotics *N* = 44 (10.1%); nucleoside analogues *N* = 15 (3.4%); ocular lubricants *N* = 7 (1.6%); non-steroidal anti-inflammatory drugs *N* = 6 (1.4%); mast-cell stabilisers *N* = 2 (0.5%); antidotes, chelators and other *N* = 1 (0.2%) and carbonic anhydrase inhibitors *N* = 1 (0.2%).

EED raters determined 40.0% (*N* = 48) of the 120 CUES referrals to EED could have been managed by an IP optometrist in primary care; 17.5% (*N* = 21) of cases could not have been managed by an IP in primary care, and in 42.5% (*N* = 51) of cases, any prescribing (or IP opinion) was N/A. Of the 40.0% of potentially manageable by an IP optometrist in primary care, the three most common diagnoses included marginal keratitis (*N* = 7, 14.6%), conjunctivitis (*N* = 6, 12.5%) and anterior uveitis (*N* = 7, 14.6%).

### Patient-Reported Experience Measures

In 2024, 6130 (11.1% of the total) PREM responses were received. Experience ratings were: 75.8% (*N* = 4644) rating their experience as 7/7 (excellent); 15.2% (*N* = 932) as 6/7; 4.4% (*N* = 267) as 5/7; 3.6% (*N* = 218) as 4/7; 0.4% (*N* = 24) as 3/7; 0.4% (*N* = 24) as 2/7 and 0.3% (*N* = 21) as 1/7. Additionally, 97.0% (*N* = 5947) of respondents indicated recommendation of the service to friends/family. Had CUES been unavailable, 41.2% (*N* = 2525) of respondents stated they would have attended their GP, and 16.8% (*N* = 1032) would have attended ED/EED.

## Discussion

### Patient Journey and Demographics

GM CUES provided care to ~55,000 patients in 2024, managing and discharging 78.2%. A further 5.2% were referred to GPs, often for further systemic testing, meaning 83.4% of cases overall were retained in primary care, comparable with Kanabar et al.’s 85.7% [12].

Many patients self-presented to CUES (69.9%), again similar to Kanabar et al.’s finding (68.7%), arguably indicating reasonable public awareness of CUES [12]. GP staff and GPs were the second- and third-most prevalent sources of attendances (11.7%, *N* = 6450 from GP staff; 6.6%, *N* = 3628 from GPs), suggesting many patients are being appropriately signposted from GPs, reducing their case load [8].

While the present study reports higher rates of HES referrals (16.6%) and face-to-face appointments (87.2%) than Kanabar et al. [12], Kanabar’s evaluation was conducted during the COVID-19 pandemic, when there was a national reduction in hospital visits [19–21]; factors likely decreasing HES referral rates and face-to-face appointments at the time. Indeed, the use of telemedicine rapidly increased during lockdown and in many settings, including CUES, this has remained [22]. When commissioned in 2020, an initial telemedicine consultation was mandated prior to those in-person, minimising face-to-face contact during the pandemic [6]. In 2022, pathway updates removed mandatory telemedicine consultations, allowing patients to proceed directly to face-to-face appointments where clinically appropriate. Thus, in 2024, only 15.7% of cases were estimated to have had a telemedicine appointment. Kanabar et al. [12] found a lower discharge rate (38.0%) after telemedicine consultations, compared to this study (88.0%), potentially owing to effective pre-screening, directing urgent cases to face-to-face appointments, leaving more benign conditions to be managed and discharged via telemedicine. Pre-screening is beneficial as CUES has a single tariff (core or enhanced for OCT/IP) per completed patient episode per practice model, meaning practices do not receive additional funding for seeing a patient for multiple appointments.

Higher proportions of attendances arose from those residing in more deprived deciles than less deprived deciles, a finding contrary to that found for uptake of England-wide routine NHS sight tests [23]. However, in the current study, the higher numbers of attendances from more deprived deciles may reflect reduced acute eyecare presentations, possibly due to concerns about expenditure on optical appliances, or simply demonstrate GM population demographics. The UK’s Office of National Statistics divides geographical units into lower-layer super output areas (LSOAs) for statistical analysis in England and Wales, and in GM, 43% of LSOAs fall within the most deprived decile [24, 25]. Further research is needed to better understand whether access to CUES in GM is equitable.

### Symptoms and Conditions

The most frequently diagnosed conditions included dry eye disease, degenerative vitreous conditions and conjunctivitis. Although not usually considered urgent or emergency, CUES is a symptom-led service, and these conditions still need urgent assessment to exclude acute presentations requiring urgent management. Evidence suggests that presenting symptoms alone are often insufficient to differentiate emergency conditions [26–28], meaning CUES appears to be an appropriate setting for assessing these patients without putting additional pressure on EED. Additionally, with the most common urgently referred conditions including retinal detachment, anterior uveitis and corneal ulcer, the current study agrees with Kanabar’s findings that the service achieves its aims of keeping more benign cases within CUES and away from EED [12].

This study found a slight change in CUES case mix with increased eyelid conditions (from 17.7 to 22.9% of total subgroup sample IP and non-IP cases) and lacrimal conditions (including all dry eye conditions) (14.0–15.9%), when compared to Kanabar et al.’s evaluation [12]. Notably, CUES eligibility is symptom-led, with investigations often required to differentiate between urgent and more benign conditions. Thus, the increased diagnoses of chronic or more benign conditions, such as blepharitis and dry eye, may indicate a return of patients’ symptom thresholds for seeking care to pre-pandemic levels and may also reflect increasing public awareness of primary care optometrists as the first point of contact for acute eye symptoms. For this comparison, Kanabar’s telemedicine data were used, as the aim was to compare presenting conditions and, under the CUES model at that time, all patients initially presented via telemedicine [12].

### Independent Prescribers

Non-IP optometrists most frequently diagnosed eyelid conditions (22.8%), while IP optometrists most frequently diagnosed conjunctival conditions (28.8%) and managed higher proportions of uveal and corneal conditions, reflecting their ability to manage more complex conditions requiring prescribing, including anterior uveitis and corneal ulcers [26]. The higher discharge rate for IP-managed cases likely reflects two factors: the effective triaging of patients as suitable for IP assessment and the higher qualification enabling IP optometrists to manage more presentations independently. The present study suggests IP optometrists manage more complex conditions and discharge more patients, increasing the scope of cases that may be retained within CUES. Similarly, in Scotland, El-Abiary et al. found that since the introduction of IP optometry, the number of primary care visits for anterior segment symptoms has increased, possibly due to IP optometrists’ ability to retain these more complex patients without referral to HES [29].

Despite the positive findings, only 0.8% of CUES cases overall were managed as IP cases in GM in 2024. HES raters categorised 40% of sampled referrals as potentially being manageable by IP optometrists, suggesting a need to expand the IP workforce to reduce HES referrals further [30].

These findings demonstrate the capacity of primary care optometry to manage patients who may have traditionally presented to or been referred to the HES. There is a meaningful opportunity at a national level to utilise primary care optometry resources better through universal access to CUES and maximising the scope of practice available. Removing barriers to IP qualification may increase further the number of patients that could be managed in primary care. These barriers include time commitment and the costs of the qualification, often self-funded [31]. Insufficient availability of hospital placements may also be problematic, with these being required to complete the IP qualification [32].

Models such as CUES enable primary care practitioners, including IP optometrists, to manage a broader range of urgent eye conditions, improving access and patient experience while reducing avoidable demand on hospital and GP services. At a national scale, wider adoption of this approach could support health system sustainability by releasing specialist capacity, improving care closer to home and aligning with NHS policy priorities to shift activity from hospital to community [33].

### Medications Prescribed

The medications most prescribed by IP optometrists were corticosteroids (43.7%), antibiotics (14.3%) and antimuscarinics (12.4%). Ocular lubricants accounted for only 1.6% of prescriptions, contrasting with existing literature, which, on a national scale, finds lubricants to be amongst the most frequently used medications [31, 34]. For example, in Wales, topical steroids, dry eye treatment, topical antibiotics and cycloplegics were the four most IP-prescribed topical medications [34]. However, in the present study, written orders were not included in the analysis, meaning ocular lubricants may be underrepresented. This finding indicates that optometrists may be appropriately providing ocular lubricants via written order, following NHS England Over The Counter (OTC) Policy recommending against routine FP10 prescribing for self-manageable diseases (such as ocular lubricants for dry eyes) as treatment is available OTC [35].

### Diagnostic Agreement

For diagnostic agreement, Kanabar et al. [12] found a 63.9% rate of agreement/partial agreement between CUES and HES diagnoses. Comparatively, this study found a higher agreement/partial agreement rate (75.0%), potentially reflecting optometrists’ growing experience within CUES and/or the increase in face-to-face assessment, which led to greater diagnostic agreement [12]. While not measurable in this analysis, diagnostic accuracy within CUES may arguably be improved through continuing education and HES referrals feedback [36].

### Patient Experience

PREM findings demonstrated high patient satisfaction regarding CUES, with 75.8% rating their experience 7/7 (excellent) and 97.0% responding positively to the Friends and Family question—a standard NHS PREM [37]. However, with an 11.1% response rate, a voluntary response bias cannot be excluded, and in the future, it may be beneficial to try to increase the response rate by using reminders, minimising questionnaire length and using mixed-mode data collection approaches [38]. Many patients reported that without CUES, they would have attended their GP or ED/EED, indicating CUES diverts cases from these medical services.

### Strengths and Limitations

This study is the largest evaluation of CUES in England to date, capturing nearly 55,000 cases, evaluating many insightful data points and addressing limitations of the previous GM CUES evaluation, including IP pathway analysis and patient satisfaction reporting [12]. Yet some limitations apply, including those resulting from the retrospective collation of data from clinical records. Outcomes were based on the last appointment, typically the clinical decision-making point regarding discharge or referral, but potentially oversimplifying how patients move through CUES. Diagnoses could be recorded as free text, meaning a subgroup was required for data cleansing feasibility, versus being able to report on 55,000 cases. As outlined, there may be ambiguities in how telemedicine versus face-to-face appointments were classified, as assumptions were made based on the clinical data recorded. This weakness in data recording has since been resolved with a 2025 IT module update. While primary care data included CUES across all GM, secondary care data were limited to MREH, potentially restricting the generalisability of secondary care outcomes. For feasibility, the referred cohort assessed was modest in necessity, and the subgroup of IP optometrists therein was limited. Other limitations include the outcomes of those not referred, i.e., false negatives were not assessed in the present evaluation, although this proportion was evaluated previously with reassuring findings [39]. Finally, cost-effectiveness was not evaluated in this study, arguably an important consideration for commissioners looking to increase the availability of IP optometrists.

## Conclusion

In 2024, CUES managed nearly 55,000 cases, retaining 83.4% in primary care. This updated evaluation shows a shift away from telemedicine towards face-to-face care, as expected post-COVID and following the removal of mandatory telemedicine appointments. CUES manages a wide range of conditions, with a large majority of cases assessed, managed and discharged without HES referral, successfully achieving its aims of diverting patients from HES and GP care. However, EEDs nationally continue to be under significant pressure, warranting further research on the cost-effectiveness of CUES and, more particularly, the role of IP optometrists. Reassessment of the false negative rate may be valuable given the introduction of IP optometrists into CUES, the case mix they may examine and the higher discharge rate associated with IP appointments reported in this study. With only 0.8% of patients managed as IP cases at present, and with an estimated 40% of the subgroup of referrals deemed potentially suitable for IP management, addressing barriers to the IP qualification, such as cost and hospital placements, appears key to improving the CUES pathway.

## Data Availability

All data for analysis and interpretation used in this work are reported within this paper.
